# Phenotypic characterization of childhood- and adult-onset food allergy among adults in the United States

**DOI:** 10.1016/j.jacig.2022.05.011

**Published:** 2022-08-12

**Authors:** Haley Hultquist, Ashley Dyer, Jialing Jiang, Ruchi Gupta, Christopher Warren

**Affiliations:** a Center for Food Allergy and Asthma Research, Institute for Public Health and Medicine, Northwestern University Feinberg School of Medicine, Chicago.; b Ann & Robert H. Lurie Children’s Hospital, Chicago.

**Keywords:** Food allergy, adult food allergy, adult-onset, prevalence, quality of life, comorbidities, health care utilization, severity

## Abstract

**Background:**

Food allergy (FA) affects ~10% of adults; however, little is known about the extent to which FA phenotypes and psychosocial burden vary depending on timing of allergy onset, whether in childhood or as an adult.

**Objective:**

This study explored FA characteristics according to timing of FA onset in US adults.

**Methods:**

Between 2015 and 2016, a cross-sectional survey was administered to 40,443 US adults. Complex survey–weighted results were tabulated across key demographic and clinical strata. Linear regression models explored covariate-adjusted variability in FA-related psychosocial burden across 3 groups: (1) adults solely with childhood-onset FA, (2) adults solely with adult-onset FA, and (3) adults with both childhood- and adult-onset FA.

**Results:**

Adults with both childhood- and adult-onset FAs, compared to adults with solely childhood- or adult-onset FAs, are significantly more likely to have severe FAs (57.3%, 52.6%, 43.2%), physician-diagnosed FAs (54.2%, 52.4%, 33.0%), and multiple FAs (74.8%, 41.0%, 30.3%) (*P* < .001). Adults solely with childhood-onset FA, compared to adults solely with adult-onset FA, had significantly lower rates of environmental allergies (27.6% vs 39.2%; *P* < .001) and medication allergies (17.3% vs 25.9%; *P* < .001). Adults with both childhood- and adult-onset FAs reported highest rates of all comorbidities. Adults solely with adult-onset FA reported significantly lower FA-related psychosocial burden (*P* < .05).

**Conclusion:**

Differences exist in reaction severity, health care utilization, atopic comorbidities, and psychosocial burden according to the timing of FA onset. Future research exploring the heterogeneity of phenotypic expressions of adult FA may inform underlying mechanisms.

IgE-mediated food allergy (IgE-FA) affects an estimated 10.8% of adults in the United States.^1^ Adult IgE-FA is an emerging public health concern,^2^ as half of these adults reported experiencing a severe reaction, and nearly 1 in 10 adults reported going to the emergency department (ED) in the past year for IgE-FA–related reactions.^1^ A recent review article reported that IgE-FA management among adults is costly as a result of higher out-of-pocket expenses and increased loss of labor productivity compared to pediatric IgE-FA.^3^ Additionally, previous research outside the United States showed that adults with IgE-FA experienced higher direct, indirect, and intangible costs than adults without IgE-FA.^4^ While IgE-FA may resolve during childhood, IgE-FA frequently persists into adulthood.^5^ In a recent nationally representative survey of over 40,000 US adults, 48.0% of adults with convincing IgE-FA developed at least 1 new IgE-FA after 18 years of age.^1^ Moreover, 25% of adults without history of childhood IgE-FA appear to be developing new allergies throughout adulthood.^6,7^

Emerging evidence indicates that phenotypic differences between childhood-onset and adult-onset IgE-FA may exist. For example, the most common allergens among adults with childhood-onset IgE-FA include egg, peanut, and milk,^8^ while the most commonly reported adult-onset allergies include shellfish, fin fish, tree nut, and peanut.^1,6,9-13^ Although previous research has examined the natural development of IgE-FA during childhood and adolescence,^14^ little research has been conducted investigating how immune tolerance to previously consumed foods is lost during adulthood. Previous studies characterizing the burden of IgE-FA among the adult population have generally not distinguished between patients with childhood- versus adult-onset IgE-FA.^10,11,15^ As such, it is unclear whether factors related to timing of IgE-FA onset—that is, in childhood or adulthood—are associated with differences in clinical atopy, demographic factors, or other phenotypic characteristics.^13^

Differences in health care utilization between adults with childhood- and adult-onset IgE-FA have also not been well examined. While research has found the estimated prevalence of physician-confirmed IgE-FA, ED visits, and current epinephrine autoinjector (EAI) prescriptions in adults with IgE-FA, these data fail to differentiate by timing of IgE-FA development.^1^ Previous research has shown that only about half of adults with IgE-FA have a physician-confirmed diagnosis, which is lower than the rate in pediatric IgE-FA.^1,8^

Additionally, it is not yet known how quality of life (QoL) differs between adults with childhood-onset IgE-FA and adult-onset IgE-FA. A recent study characterized the psychosocial burden of IgE-FA among a population-based sample of US adults and found specific demographic characteristics and allergic conditions to be associated with increased psychosocial burden.^16^ While this study suggested that certain aspects of health care utilization, such as EAI prescriptions and ED visits, were associated with a greater IgE-FA–related psychosocial burden among the population of adults with IgE-FA,^16^ the research remains mixed. One study exploring specific factors that affect QoL in children and adults with IgE-FA found no association between current EAI prescriptions and QoL impairment.^17^

To better understand the phenotypic characterization of childhood- and adult-onset IgE-FA among adults in the United States, we aimed to characterize the distribution and determinants of IgE-mediated FA in 3 US adult subpopulations: (1) adults with solely childhood-onset IgE-FA, (2) adults with solely adult-onset IgE-FA (over 18 years of age), and (3) adults with both childhood- and adult-onset IgE-FA. Utilizing convincing symptom reports, likely cases of IgE-mediated FA were identified in these 3 subpopulations. We sought to explore differences in sociodemographic predictors, IgE-FA severity, reaction characteristics, health care utilization, atopic comorbidities, and IgE-FA–related psychosocial burden among these 3 groups (Fig 1).

## METHODS

Eligible study participants included adults (≥18 years old) who resided in a US household and were able to complete the survey in English or Spanish online or by telephone. Similar to the 2009-2010 national pediatric IgE-FA prevalence survey,^18^ this study relied on a nationally representative household panel to support population-level inference. Study participants were first recruited from NORC at the University of Chicago’s, nationally representative, probability-based AmeriSpeak panel. NORC is a private independent survey research organization, founded in 1941 as the National Opinion Research Center. Respondent weights were reconciled with external population totals associated with age, sex, education, race and ethnicity, housing tenure, telephone status, and census division via iterative proportional fitting to improve external validity. To increase the precision of population parameter estimates, the population-weighted AmeriSpeak responses were also augmented via iterative proportional fitting with calibration-weighted, non–probability-based responses obtained through Survey Sampling International. The complex survey development, sampling, weighting, and analysis procedures utilized for the current study have been previously detailed.^1^

### Outcome measures

Reported FAs were only considered to be convincingly IgE mediated if the most severe reaction reported to that food included at least 1 symptom on the stringent symptom list developed by our expert panel, even if IgE-FAs were reported to be physician diagnosed (see Fig E1 in this article’s Online Repository at www.jaci-global.org). The “convincing IgE-FA” definition is intended to identify cases of IgE-mediated FA. Timing of allergy onset and outgrowing an allergy (if applicable) were assessed for each specific allergy. Childhood-onset IgE-FA is defined as having onset of the first food allergen-specific reaction before age 18. Convincing IgE-FAs for which a doctor’s diagnosis was also reported were considered to be physician confirmed. A severe reaction history was indicated by report of multiple specific stringent symptoms occurring within 2 or more of 4 organ systems (skin/oral mucosa, gastrointestinal, cardiovascular, and respiratory) in response to the question, “Think back to the most severe allergic reaction to … that you have ever had. What were your symptoms?”

Both current and outgrown IgE-FAs were assessed. A complete description of the survey development, testing, and categorization of allergy type has been previously published.^1^ The Food Allergy Independent Measure (FAIM) was administered to all respondents reporting a current IgE-FA. This validated measure contains 6 questions, which are scored on a 1-to-7–point scale, with higher scores indicating greater psychosocial burden and lower FA-related QoL.^19^

### Statistical analysis

Complex survey–weighted means and proportions were calculated to estimate most quantities of interest including IgE-FA prevalence and frequency of specific demographic and clinical characteristics among sampled US adults by Stata 17 software (StataCorp, College Station, Tex) using ‘svy: commands.’ Relative proportions of demographic characteristics were compared using weighted Pearson chi-square statistics, which were corrected for the complex survey design with the second-order correction of Rao and Scott and converted into *F* statistics. Covariate-adjusted complex survey–weighted logistic regression models compared relative prevalence and other assessed IgE-FA outcomes by participant characteristics. Linear regression models explored covariate-adjusted variability in IgE-FA–related psychosocial burden. Two-sided hypothesis tests were used; 2-sided *P* < .05 was considered statistically significant.

## RESULTS

### Demographic distribution of adults with IgE-FA

Surveys were completed by 40,443 adults living in the United States. A survey completion rate of 51.2% (7,218 responses of 14,095 invitees) was observed from the population-based AmeriSpeak panel, from which prevalence was estimated. As reported in Table I, demographic characteristics of the weighted sample were representative of the US general adult population. Among adults with IgE-FA, 51.9% (95% confidence interval [CI], 50.2-53.7) had solely childhood-onset IgE-FA, 21.1% (95% CI, 19.8-22.5) had solely adult-onset IgE-FA, and 27.0% (95% CI, 25.4-28.7) had both childhood- and adult-onset IgE-FA (Table I).

**FIG 2.**
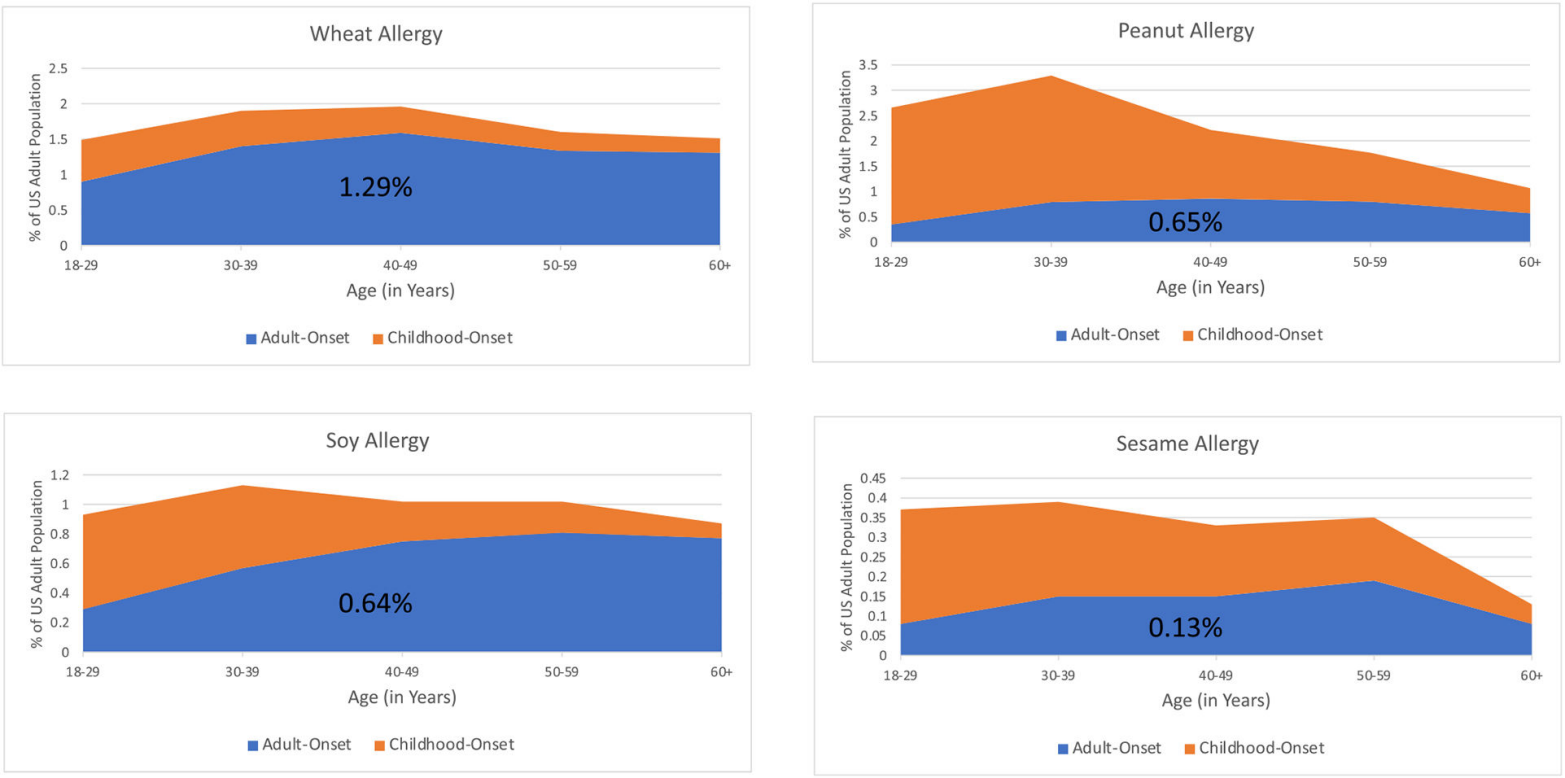
Prevalence of wheat, peanut, soy, and sesame among US adults by timing of FA onset. *Orange shading* represents prevalence of adults with childhood-onset of specific FA at each given age range; *blue shading,* prevalence of adults with adult-onset of specific FA at each given age range.

These survey data indicate that although there is substantial heterogeneity among US adults with IgE-FA regarding the typical timing of IgE-FA onset, older people are more likely to report the onset of each IgE-FA during adulthood. However, some of these specific IgE-FAs are far more likely to start during adulthood (eg, wheat, soy), while others are more likely to start during childhood (eg, peanut, sesame) (Fig 2; and see Fig E2 in the Online Repository at www.jaci-global.org).

### Characteristics and severity of adult IgE-FA

Adults with solely childhood-onset IgE-FA were more likely to report a history of ≥1 severe food-allergic reactions (52.6% vs 43.2%, *P* < .001), physician-diagnosed IgE-FA (52.4% vs 33.0%, *P* < .001), and multiple IgE-FAs (41.0% vs 30.3%, *P* < .001) compared to adults with solely adult-onset IgE-FA. Adults with both childhood- and adult-onset IgE-FA were also more likely to report a history of ≥1 severe food-allergic reactions (57.3%), physician-diagnosed IgE-FA (54.2%), and multiple IgE-Fas (74.8%) than either group individually (Table II). This trend in adult IgE-FA was consistent across the 9 most common food allergens with adults with solely childhood-onset IgE-FA reporting higher rates than adults with solely adult-onset IgE-FA (see Table E2 in the Online Repository at www.jaci-global.org).

Adults with solely childhood-onset IgE-FA also reported significantly higher rates of current EAI prescriptions (28.2% vs 18.1%, *P* < .001), 1 or more lifetime ED visits (44.9% vs 23.1%, *P* < .001), and 1 or more allergy-related ED visits in the past year (9.5% vs 4.9%, *P* < .001) compared to adults with adult-onset IgE-FA. This trend also remained consistent across the 9 common food allergens, with childhood-onset rates being higher than adult-onset rates (Table E2).

In general, adults with solely adult-onset IgE-FA reported significantly lower rates of diagnostic testing than adults who had IgE-FA onset in childhood (Table II).

Specific FA reaction symptoms respondents reported experiencing during their most severe food-allergic reaction differed greatly between adults with childhood-onset and adult-onset IgE-FA. Across the 9 most common food allergens, adults with childhood-onset IgE-FA reported a higher frequency of stringent skin, respiratory, gastrointestinal, and cardiovascular symptoms than the adults with the corresponding adult-onset IgE-FA. Even when analyzing the most prevalent IgE-FAs among adults (shellfish, finfish, tree nuts, and peanut), adults that developed the same IgE-FA during childhood were more likely to report severe reaction symptoms categorized (see Table E1 in the Online Repository at www.jaci-global.org).

### Comorbid conditions among adults with IgE-FA

Among adults with IgE-FA, the prevalence of specific physician-diagnosed comorbid conditions varied considerably depending on the timing of IgE-FA onset. Adults with solely childhood-onset IgE-FA, compared to adults with solely adult-onset IgE-FA, had higher rates of asthma and atopic dermatitis. Comparatively, adults with solely adult-onset IgE-FA, compared to adults with solely childhood-onset IgE-FA, had higher rates of environmental allergies, medication allergy, insect sting allergy, and latex allergy. Adults with both childhood- and adult-onset IgE-FA reported the highest rates of all atopic comorbidities assessed by the survey. Survey data showed that respondents with greater numbers of comorbid atopic conditions were more likely to have adult-onset IgE-FA. Conversely, an increase in comorbid conditions was associated with decreased odds of an adult having solely adult-onset IgE-FA (Table III).

### QoL among adults with IgE-FA

**FIG 3.**
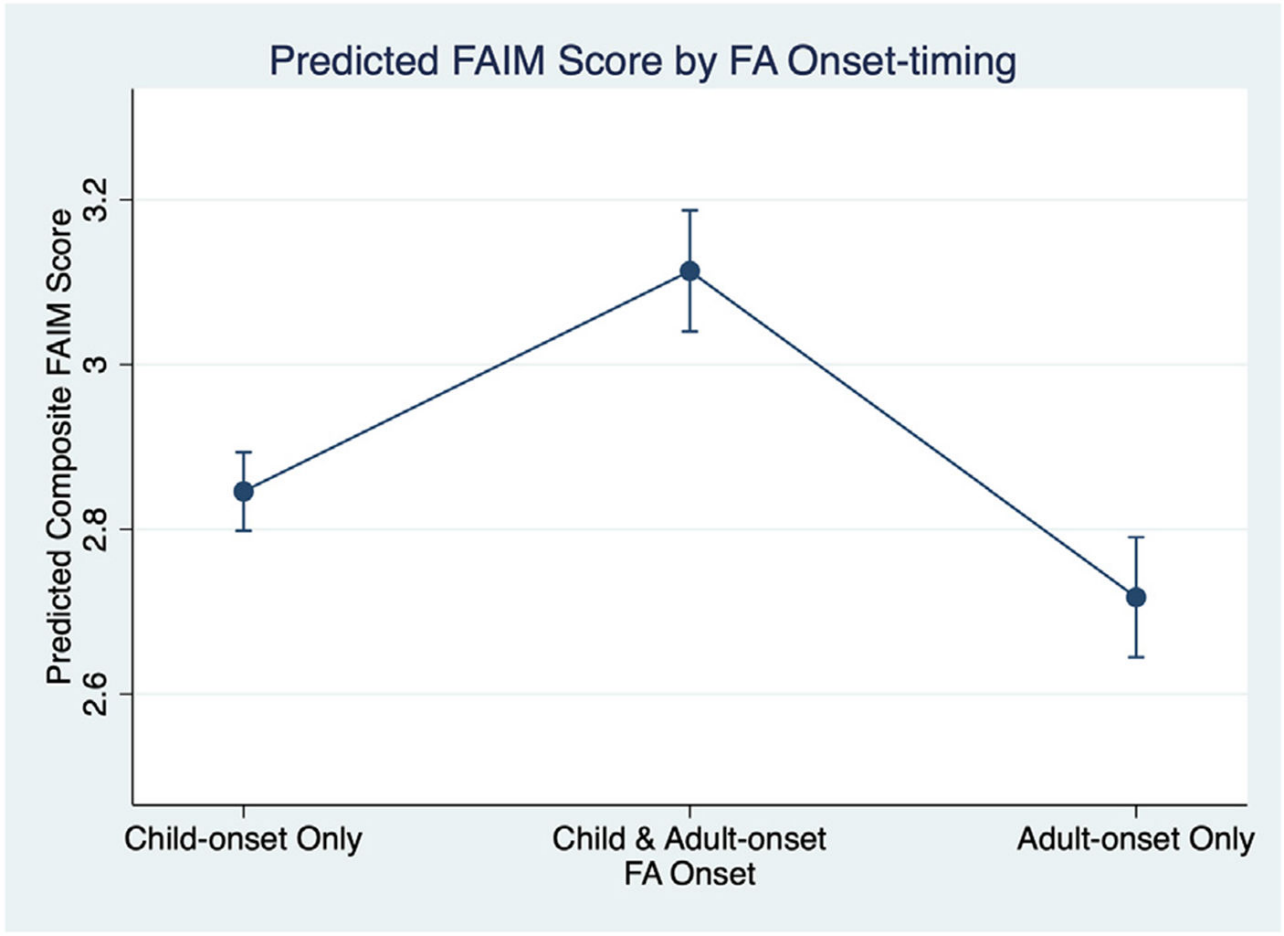
Model-predicted estimated mean Food Allergy Independent Measure (FAIM) scores and corresponding 95% CIs for US adults with only childhood-onset IgE-FA, US adults with both childhood-onset and adult-onset IgE-FAs, and US adults with adult-onset IgE-FA.

After adjustment for demographic and atopic disease characteristics, there were differences in QoL impairment for adults with IgE-FA, depending on timing of IgE-FA onset. Specifically, adults with solely adult-onset IgE-FA reported lower levels of QoL impairment compared to adults with solely childhood-onset IgE-FA and adults with both childhood- and adult-onset IgE-FA (Fig 3).

## DISCUSSION

To our knowledge, this is one of the first studies to describe IgE-FA distribution and determinants among (1) adults with solely childhood-onset IgE-FA, (2) adults with solely adult-onset IgE-FA, and (3) adults with both childhood- and adult-onset IgE-FA; as such, it provides data that help articulate differences in sociodemographic predictors, IgE-FA severity, reaction characteristics, health care utilization patterns, atopic comorbidities, and IgE-FA–related psychosocial burden according to timing of IgE-FA onset. The estimated 14 million US adults with solely childhood-onset IgE-FA reported higher frequency of severe symptoms than the estimated 5.8 million adults with only adult-onset IgE-FA. Adults with childhood-onset IgE-FA had higher rates of health care utilization, with more than half having a physician-confirmed IgE-FA diagnosis, compared to only 30% of adults with adult-onset IgE-FA. The adults with childhood-onset IgE-FA also reported higher QoL impairment than adults with solely adult-onset IgE-FA.

The demographic data from this study revealed sex differences in age-specific IgE-FA: men had a higher prevalence of IgE-FA in all 3 categories of adults with IgE-FA, but women had a higher risk of developing adult-onset IgE-FA. However, the demographic characteristics associated with pediatric IgE-FA, such as race and ethnicity, were not directly associated with the risk of adult-onset IgE-FA. The demographic characteristics of each group of adults with IgE-FA align with previous research indicating that childhood-onset IgE-FA is more prevalent among male subjects, and that female subjects have a higher risk of developing IgE-FA during adulthood.^6,8,11,12^ Among adults with IgE-FA, older adults (40-60+ years) with IgE-FA were more likely to have developed IgE-FA during adulthood. This is consistent with previous findings that IgE-FA onset may occur at any time during adulthood, and that IgE-FA tends to persist once developed.^6,9,12^ In the present cross-sectional study, race and ethnicity, income, and age were not predictive of adult-onset IgE-FA. However, existing research demonstrates that these demographic factors may affect the incidence of childhood-onset IgE-FA in children, in that IgE-FA is more prevalent among self-reported non-Hispanic Black children than non-Hispanic White children, and is more prevalent in male than female children.^8^ Establishment of, and clinical follow-up within, longitudinal cohorts of patients with adult-onset IgE-FA would provide further insight into the natural history, determinants, and correlates of adult-onset IgE-FAs.

Adults with solely childhood-onset IgE-FA were more likely to report experiencing severe symptoms, such as chest tightening or vomiting, compared to adults who developed the same IgE-FA during adulthood. The current literature describes how children with peanut, tree nut, or shellfish allergies report some of the most severe IgE-FA–related reaction symptoms,^20^ and the present survey data also suggest a similar trend in adults with childhood-onset IgE-FA. With these known severe allergies persisting into adulthood,^11,13^ the findings corroborate existing data demonstrating that these foods can continue to elicit severe reaction symptoms in adults.^20^ Additionally, the period of adolescence and young adulthood provides unique insight into how symptoms related to childhood-onset IgE-FA persist into adulthood and this period of life should be further investigated to better understand symptoms and reaction severity experienced by adults with childhood-onset IgE-FA. While adults with adult-onset IgE-FA did not report the high severity of reactions as the adults with childhood-onset IgE-FA, understanding the specific reaction symptoms and typical patterns of allergy resolution within adults with IgE-FA is critical to improving our understanding of IgE-FA disease progression over the life-span. In turn, this can provide patients and their clinical care providers with more actionable information for making informed management and treatment decisions.

IgE-FA–related health care utilization was significantly higher for adults with solely childhood-onset IgE-FA compared to those with adult-onset IgE-FA. This includes higher rates of physician-confirmed IgE-FA diagnosis, EAI prescriptions, diagnostic testing, and ED visits, all of which support previous research showing that adults with adult-onset IgE-FA have lower levels of health care usage.^16^ While this variation in health care usage may reflect trends in severity (real or perceived), it also may be affected by the low rate of adults with a convincing IgE-FA who have a physician-diagnosed IgE-FA.^1^ Because only approximately half of adults with IgE-FA received a physician-confirmed IgE-FA diagnosis,^1^ it is possible that the low levels of health care utilization among adults with adult-onset IgE-FA are related to a lack of patient knowledge of IgE-FA diagnosis and management, a lack of accessibility/availability to specialty care, or a lack of effective communication between patient and provider regarding symptom severity. Individuals with a physician-confirmed IgE-FA diagnosis are likely to receive guidance on proper IgE-FA management at the time of diagnosis, including prescription of an EAI, the only first-line treatment for anaphylaxis. It remains important for adults who perceive themselves to be food allergic to seek clinical confirmation of allergy. Targeted clinical allergy testing often leads to a more specific diagnosis (eg, allergy to raw egg, but not egg in cooked forms) and reduce unnecessary food avoidance and unnecessary heightened vigilance.

While our findings suggest that adults with adult-onset IgE-FA do not report high levels of allergist care, the survey data from adults with childhood-onset IgE-FA suggest that efforts to improve access to allergist care alone may be insufficient for improving IgE-FA management among the broader adult population with IgE-FA from a psychosocial perspective. Adults with solely childhood-onset IgE-FA had greater health care utilization than adults with solely adult-onset IgE-FA. Direct allergy care and hypervigilant management may be contributing to lower QoL because the group of adults with childhood-onset IgE-FA reported the greatest burden of disease. Previous literature describing the effect of EAI prescriptions and diagnostic testing (skin prick tests, oral food challenges) on psychosocial well-being show that there exists an association with high levels of stress and anxiety.^16,21-23^ However, the research is mixed. A study by Saleh-Langenberg et al^17^ looked at factors that may be predictive of IgE-FA–related QoL in a population of 404 adults with IgE-FA and found that proper allergy diagnosis and EAI prescriptions did not contribute to IgE-FA–related psychosocial burden. The effect of health care utilization on adult IgE-FA–related psychosocial burden should be further explored.

Our finding that an increased number of comorbid conditions resulted in increased odds of an adult having childhood-onset IgE-FA onset aligns with research that describes a strong relationship between childhood-onset IgE-FA and specific atopic comorbid conditions, such as asthma and atopic dermatitis.^8,24-26^ This pattern of developmentally staged atopic disease co-occurrence is often characterized as an “atopic march”—a progression of atopic pathology where children with atopic dermatitis are likely to develop additional atopic conditions over time through cutaneous sensitization, ultimately resulting in greater susceptibility for developing childhood-onset IgE-FA.^14,27^ While some existing literature highlights associations between the presence of asthma or eczema and new food sensitization in adulthood, our data only identified this association in adults with childhood-onset IgE-FA.^10,12,28^ However, among adults with solely adult-onset IgE-FA, the prevalence of comorbidities reinforced the findings of previous research suggesting a link between allergic rhinitis and adult-onset IgE-FA and other research suggesting a link between latex allergy and adult-onset IgE-FA.^6,11^ Other factors known to increase risk of adult-onset IgE-FA and/or reduce thresholds of clinical reactivity include alcohol consumption, receipt of antacids or nonsteroidal anti-inflammatory drugs, and hormone changes.^6,9,11,29,30^ These findings in reported comorbidities and related factors suggest that differences may exist in the underlying mechanisms that cause development of childhood- versus adult-onset IgE-FA. Future longitudinal studies should assess both atopic and nonatopic contributors to IgE-FA development during adulthood to better understand the mechanisms contributing to adult food sensitization and tolerance. Specifically, we need additional knowledge on adult-onset IgE-FA to better differentiate between adult-onset IgE-FA, pollen FA syndrome, and other secondary allergies, with respect to both their putative phenotypic and endotypic variability.

Adults with both childhood-onset and adult-onset IgE-FA reported the highest effect of IgE-FA on QoL, even after accounting for key sociodemographic and clinical allergy characteristics. These adults have likely faced certain known factors of living with IgE-FA associated with diminished QoL for the longest period of time, such as constant allergen avoidance, fear of allergic reaction, and diagnostic testing.^16,31^ Also, the group of adults with both childhood- and adult-onset IgE-FA is dealing with allergies that developed in 2 different phases of life, childhood and adulthood, and the resulting heightened burden may suggest a lack of effective support for IgE-FA management for this lengthy time frame. Adults with childhood-onset IgE-FA had to manage their IgE-FA during the transition from childhood into adulthood, through the challenging period of shifting from dependent to independent medical care. This transition is known to be stressful and difficult, especially for people with IgE-FA, who must undergo the shift from having their chronic disease managed by a parent or caregiver to self-management and may result in the worsening of chronic conditions.^32,33^ Further exploration of IgE-FA onset timing’s effect on patient QoL is warranted to inform appropriate management strategies.

### Limitations

With population-based surveys, there are some notable limitations, specifically related to likely self-reporting and recall bias relating to reported allergic reaction symptomatology and its attribution to specific perceived causative foods. Because the current reference standard of IgE-FA diagnosis is a double-blind placebo-controlled food challenge, which is not feasible for a population-based survey, stringent criteria regarding timing and type of symptoms were utilized to screen for the presence of convincing IgE-FA. As such, it is possible that some individuals identified as having “convincing IgE-FA” in the present study may in fact have distinct, non–IgE-mediated conditions—or none at all—and conversely, that some individuals with true IgE-mediated allergy may not be captured by our algorithm. However, by using the present “convincing IgE-FA” case definition, we are assessing the subpopulation of US adults who either truly do have IgE-FA or who do not actually have IgE-FA but are living their daily lives as if they do, and therefore experiencing the associated psychosocial burden.^16^

### Conclusion

Among the 3 groups of adults (adults with solely childhood-onset IgE-FA, adults with solely adult-onset IgE-FA, and adults with both childhood- and adult-onset IgE-FA), there were differences in reaction severity, health care utilization, comorbid conditions, and QoL. In general, adults with childhood-onset IgE-FA reported more severe IgE-FA–related reaction symptoms and higher rates of physician-confirmed diagnoses and EAI prescriptions. The differences in reported comorbid conditions suggest that there may be differing mechanisms of IgE-FA development between childhood- and adult-onset IgE-FA. Overall, the phenotypic variances that are based on timing of IgE-FA onset reveal that tailored management strategies may be necessary in addition to the need for guideline-informed care, including physician diagnoses and EAI prescriptions. The age at which IgE-FA develops needs to be accounted for in the creation of treatment plans for adults with IgE-FA. As a result of these phenotypic differences, future research should further articulate possible mechanisms contributing to solely adult-onset IgE-FA because the current data highlight different factors than those known to be associated with solely childhood-onset IgE-FA.

## Supplementary Material

1

## Figures and Tables

**FIG 1.**
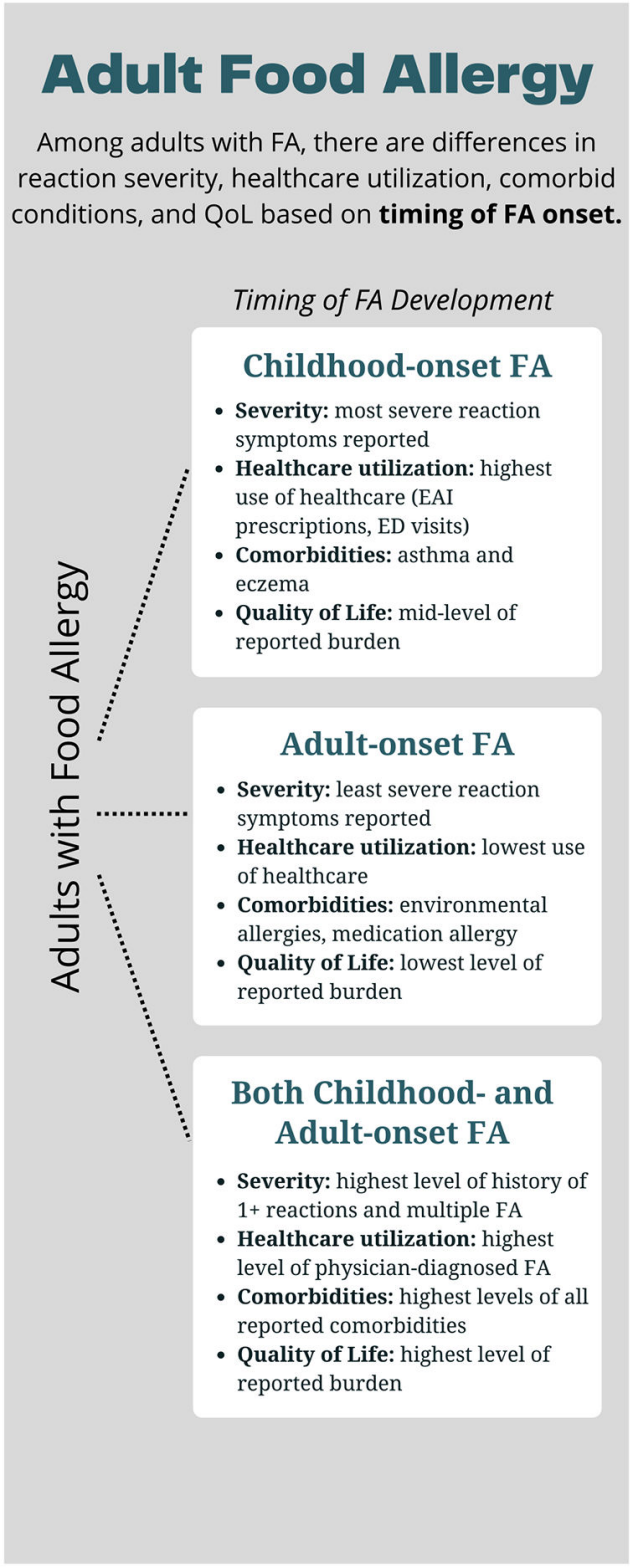
Summary of study findings.

**TABLE I.** Demographic distribution of adults with and without FA by onset timing

	US adults		Adults with convincing FA		Adults with only childhood-onset FA		Adults with only adult-onset FA		Adults with both childhood-onset and adult-onset FA		
Variable	Point estimate (%)	95% CI	Point estimate (%)	95% CI	Point estimate (%)	95% CI	Point estimate (%)	95% CI	Point estimate (%)	95% CI	2-sided *P* value*
Race/ethnicity											
Asian, non-Hispanic	3.9	3.6-4.1	4.1	3.5-4.8	4.6	3.8-5.7	4.3	3.2-5.9	3.0	2.1-4.3	<.001
Black, non-Hispanic	11.7	11.3-12.1	12.2	11.1-13.3	12.8	11.2-14.5	14.6	12.1-17.5	9.1	7.4-11.1	
White, non-Hispanic	64.9	64.2-65.6	61.1	59.3-62.9	57.2	54.7-59.6	60.2	56.6-63.8	69.5	65-9-72.9	
Hispanic	15.5	14.9-16.1	16.6	15.2-18.2	19.2	17.1-21.4	15.2	12.5-18.3	12.6	9.9-15.9	
Multiple/other	4.1	3.8-4.4	6.0	5.1-7.1	6.3	4.9-8.0	5.6	4.2-7.6	5.8	4.2-7.9	
Sex											
Female	51.7	51.0-52.4	66.4	64.8-68.1	61.2	58.8-63.5	73.6	70.4-76.5	71.4	68.0-74.6	<.001
Male	48.3	47.6-49.0	33.6	31.9-35.2	38.8	36.5-41.2	26.5	23.6-29.6	28.6	25.4-32.1	
Age											
18-29 years	21.5	20.8-22.1	22.6	21.1-24.1	32.8	30.6-35.2	14.0	11.9-16.4	9.2	7.3-11.7	<.001
30-39 years	17.0	16.5-17.5	20.1	18.7-21.5	22.1	20.1-24.1	18.8	16.2-21.7	17.1	14.5-20.1	
40-49 years	16.8	16.3-17.3	15.6	14.4-16.9	14.9	13.3-16.6	17.8	15.3-20.7	15.5	13.1-18.3	
50-59 years	18.0	17.5-18.5	19.9	18.5-21.4	16.3	14.6-18.1	22.3	19.4-25.5	25.0	21.9-28.4	
60+ years	26.8	26.2-27.4	21.9	20.4-23.3	13.9	12.4-15.7	27.1	23.9-30.5	33.2	29.9-36.6	
Household income, US$											
<25,000	16.6	16.2-17.1	16.3	15.2-17.6	16.9	15.1-18.8	16.8	14.5-19.3	15.0	13.0-17.4	.84
25,000-49,999	22.0	21.4-22.5	22.2	20.9-23.6	21.1	19.4-22.9	23.7	20.8-26.7	23.2	20.5-26.1	
50,000-99,999	30.9	30.3-31.5	33.4	31.8-35.0	33.3	31.2-35.5	32.9	29.6-36.4	33.8	30.4-37.4	
100,000-149,999	19.6	19.0-20.2	19.1	17.6-20.8	19.4	17.3-21.7	18.1	15.2-21.4	19.4	16.4-22.8	
>150,000	10.9	10.4-11.5	8.9	7.9-10.1	9.3	7.9-11.0	8.5	6.6-11.0	8.6	6.6-11.1	
Insurance status (AmeriSpeak only)											
Uninsured	7.6	6.5-8.7	8.7	5.9-12.5	10.6	6.6-16.7	7.5	3.7-14.8	6.7	2.6-16.4	.61
Private	64.4	62.5-66.2	61.8	56.5-66.8	62.4	54-3-69-8	57.3	47.4-66.6	64.2	54.2-73.1	
Public	28.1	26.4-29.8	29.5	25.0-34.5	27.0	20.7-34.5	35.2	26.4-45.1	29.1	21.2-38.6	
Census division											
New England	4.9	4.5-5.1	4.7	4.0-5.5	4.2	3.4-5.2	3.9	2.8-5.6	6.1	4.5-8.2	.33
Middle Atlantic	13.5	13.0-14.0	14.4	13.1-15.9	14.6	12.8-16.5	14.4	11.7-17.7	14.1	11.3-17.4	
East North	13.2	12.8-13.6	12.5	11.5-13.6	12.5	11.1-14.1	13.1	11.0-15.5	12.0	10.1-14.3	
West North	8.4	8.1-8.8	8.1	7.2-9.1	8.3	7.1-9.6	7.6	6.0-9.6	8.1	6.4-10.3	
South Atlantic	6.0	5.6-6.3	20.3	19.0-21.7	19.2	17.5-21.0	22.1	19.3-25.1	21.1	18.4-24.2	
East South	6.0	5.6-6.3	5.8	5.1-6.7	6.5	5.4-7.8	5.9	4.4-7.8	4.6	3.5-6.1	
West South	11.7	11.3-12.2	10.4	9.3-11.6	10.4	8.9-12.2	9.1	7.2-11.5	11.2	9.1-13.7	
Mountain	7.2	6.9-7.6	8.4	7.4-9.4	7.7	6.5-9.2	9.4	7.5-11.7	8.8	6.9-11.1	
Pacific	15.1	14.6-15.7	15.5	14.2-16.9	16.7	14.8-18.7	14.5	12.2-17.2	14.0	11.7-16.6	

* Two-sided *P* value for *F* statistic corresponding to second-order Rao-Scott survey-adjusted chi-square.

**TABLE II.** Characteristics of adults with FA by onset timing

Variable	Proportion of adults with childhood- onset *only* FA with:	Proportion of adults with childhood *and* adult-onset FA with:	Proportion of adults with adult- onset *only* FA with:	2-sided *P* value*
Severe convincing FA†	52.6 (50.2-55.0)	57.3 (53.7-60.8)	43.2 (39.6-46.8)	<.001
Physician-diagnosed FA	52.4 (49.9-54.8)	54.2 (50.6-57.8)	33.0 (29.8-36.4)	<.001
Multiple FAs	41.0 (38.7-43.4)	74.8 (71.6-77.9)	30.3 (27.1-33.7)	<.001
Current epinephrine prescription	28.2 (26.3-30.3)	25.6 (22.8-28.7)	14.1 (12.0-16.6)	<.001
One or more lifetime ED visits	44.9 (42.5-47.3)	41.5 (38.0-45.1)	23.1 (20.4-26.1)	<.001
One or more FA-related ED visits in past year	9.5 (8.2-11.0)	10.9 (8.8-13.5)	4.9 (3.6-6.7)	<.001
Allergy diagnosed via skin prick test	35.0 (32.9-37.2)	37.8 (34.4-41.4)	17.0 (14.7-19.6)	<.001
Allergy diagnosed via blood test	21.0 (19.2-22.9)	21.9 (19.2-24.9)	13.0 (10.9-15.5)	<.001
Allergy diagnosed via oral food challenge	14.6 (13.2-16.2)	15.7 (13.3-18.5)	8.1 (6.2-10.4)	<.001

Data are shown as percentage population-weighted frequency and 95% CI.

* Two-sided *P* value for *F* statistic corresponding to second-order Rao-Scott survey-adjusted chi-square.

† Severe convincing FA defined by history of ≥1 severe food allergic reactions.

**TABLE III.** Prevalence of physician-diagnosed comorbid conditions among adults with FA by time of onset

	Proportion of adults with childhood-onset *only* FA who have:		Proportion of adults with childhood *and* adult-onset FA who have:		Proportion of adults with adult- onset *only* FA who have:		
Variable	Point estimate (%)	95% CI	Point estimate (%)	95% CI	Point estimate (%)	95% CI	2-sided *P* value*
Asthma	24	22.0-26.2	27	24.1-30.2	20.8	18.1-23.7	.02
Atopic dermatitis/eczema	12.3	10.7-13.9	14	11.7-16.7	9.8	7.8-12.2	.04
Environmental allergies	27.6	25.5-29.7	44.7	41.1-48.3	39.2	35.7-42.8	<.0001
Insect sting allergy	6.4	5.4-7.7	12.4	10.2-15.0	8	6.3-10.1	<.0001
Latex allergy	4.3	3.5-5.2	11.3	9.1-13.9	5.8	4.4-7.6	<.0001
Medication allergy	17.3	15.5-19.2	34	30.7-37.5	25.9	22.9-29.1	<.0001

* Two-sided *P* value for *F* statistic corresponding to second-order Rao-Scott survey-adjusted chi-square.

## Clinical implications:

Among adults with FA, there are differences in reported reaction severity, health care utilization, comorbid conditions, and QoL according to the timing of FA onset, child or adult.

## Abbreviations used

CI: Confidence interval
EAI: Epinephrine autoinjector
ED: Emergency department
FA: Food allergy
IgE-FA: IgE-mediated FA
QoL: Quality of life
