# Supplementary material for: Phenotypic characterization of childhood- and adult-onset food allergy among adults in the United States
Source: J Allergy Clin Immunol Glob. 2022 Aug 12;1(4):257–64. doi: 10.1016/j.jacig.2022.05.011 (PMC9683432; doi:10.1016/j.jacig.2022.05.011)
Supplement: Supplementary Material [file mmc1.docx]

**eFigure 1.**List of Stringent Reaction Symptoms used to Categorize Convincingly IgE-mediated Food Allergy


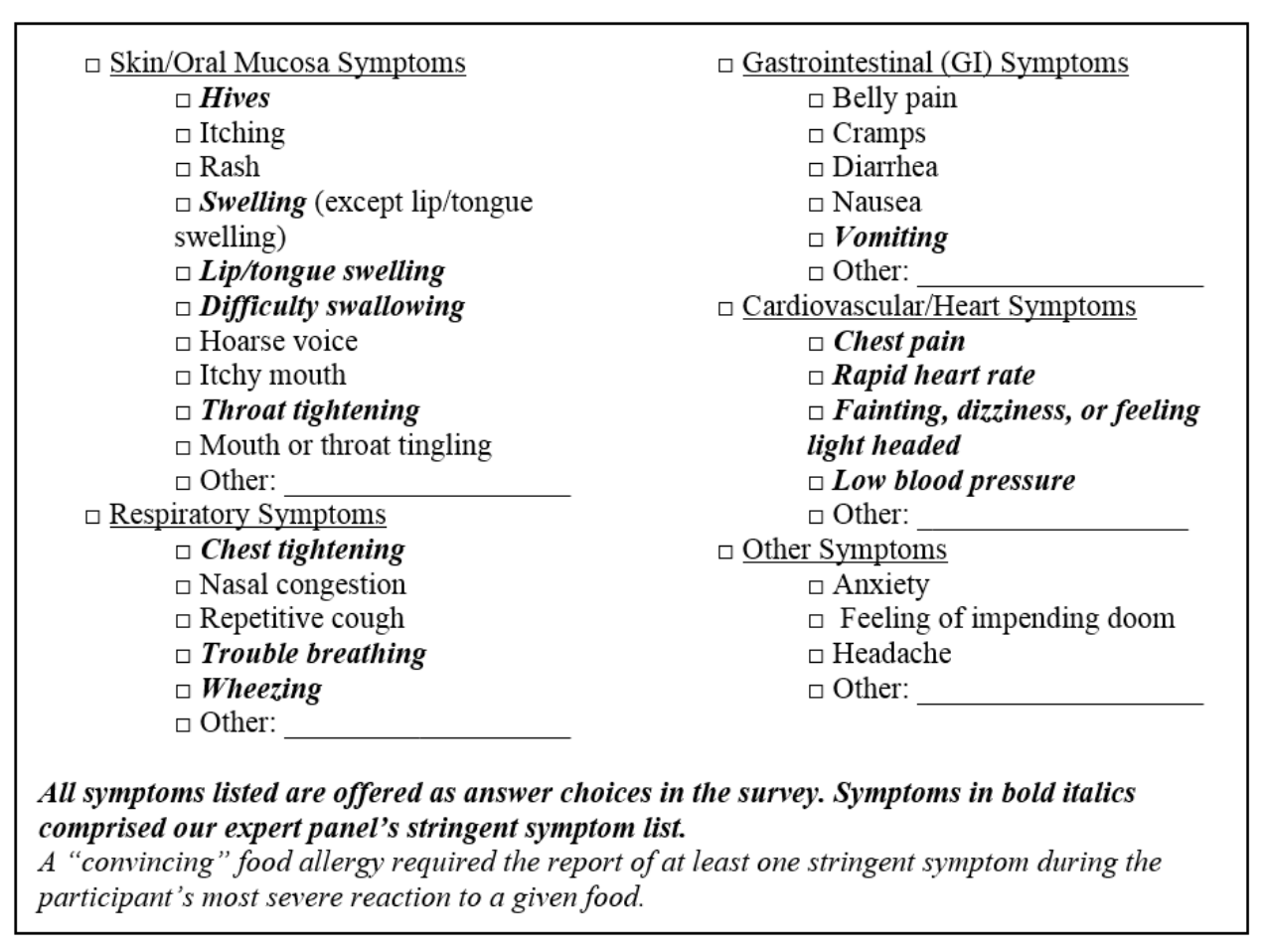


**eFigure 2.** Prevalence of Egg, Tree Nut, Shellfish, Milk, and Finned Fish Allergy in Adults Based on Timing of Onset

**
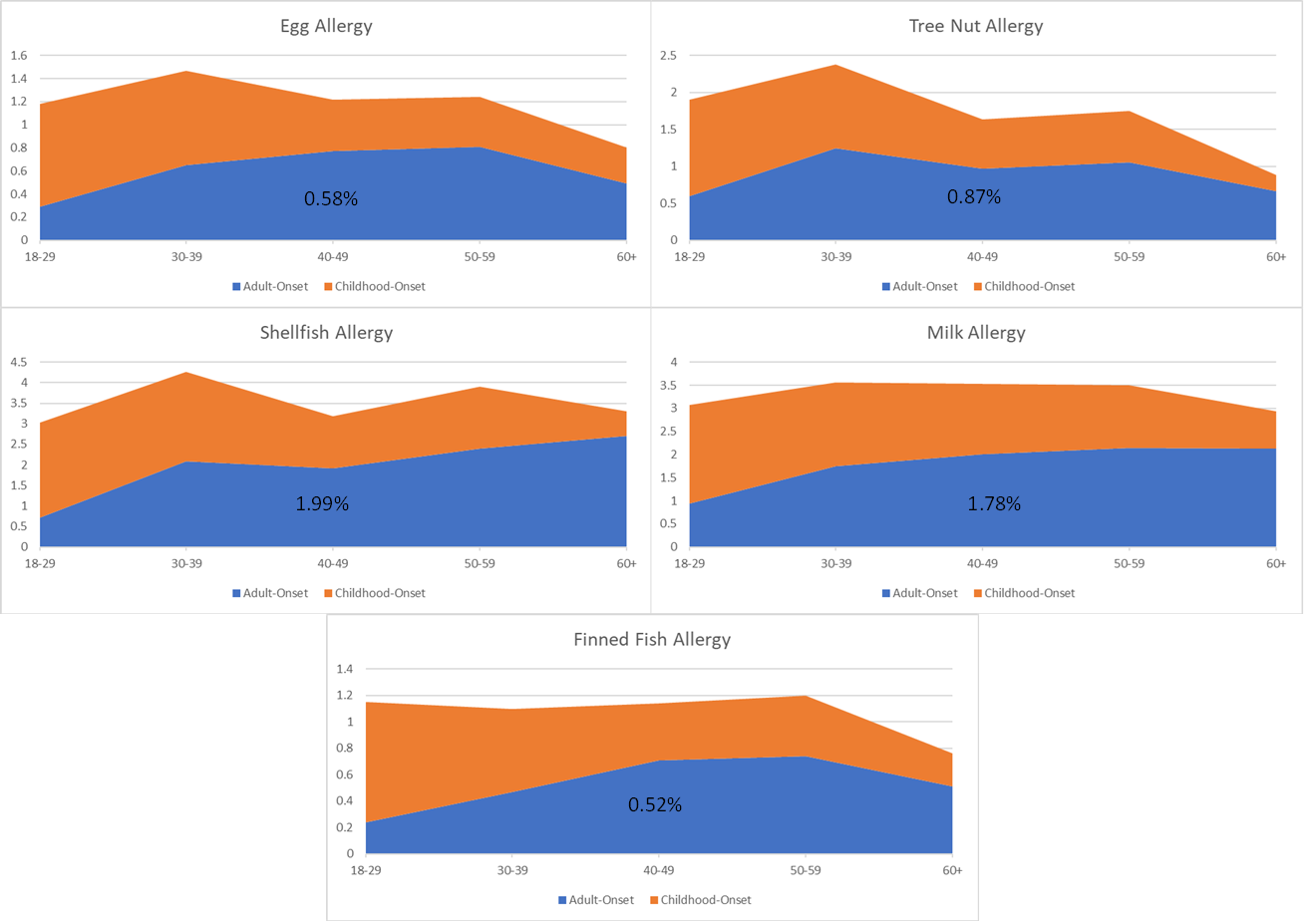
**

**eTable 1.** Symptom Characteristics of Adults with Childhood-Onset vs. Adult-Onset “Top Nine” Food Allergens, Population-weighted Frequency % (95% CI)

|  | **Symptom Characteristics of Adults with Childhood-Onset vs. Adult-Onset Peanut Allergy, Population-weighted Frequency % (95% CI)** | | | | | **Symptom Characteristics of Adults with Childhood-Onset vs. Adult-Onset Treenut Allergy, Population-weighted Frequency % (95% CI)** | | | | |
| --- | --- | --- | --- | --- | --- | --- | --- | --- | --- | --- |
| **Symptoms** | **Childhood Onset Peanut Allergy** | | **Adult Onset Peanut Allergy** | | **p value** | **Childhood Onset Treenut Allergy** | | **Adult Onset Treenut Allergy** | | **p value** |
|  | **Point Estimate** | **(95% CI)** | **Point Estimate** | **(95% CI)** |  | **Point Estimate** | **(95% CI)** | **Point Estimate** | **(95% CI)** |  |
| **Skin/Oral/Mucosal Tissue** |  |  |  |  |  |  |  |  |  |  |
| **Any *Stringent* Skin** | 75.1 | 70.9-78.9 | 67.9 | 59.0-75.7 | 0.11 | 81.3 | 76.2-85.5 | 77.6 | 70.2-83.6 | 0.37 |
| *Hives* | 63.2 | 58.8-67.3 | 28.3 | 22.7-34.8 | 0 | 73.7 | 68.3-78.5 | 31 | 25.6-37.0 | 0 |
| Itching | 68.5 | 64.7-72.2 | 25.6 | 20.3-31.7 | 0 | 81.7 | 77.2-85.5 | 33.8 | 28-40.1 | 0 |
| *Rash* | 75.7 | 72.2-79.0 | 27.8 | 22.3-34.0 | 0 | 57.7 | 51.9-63.4 | 23 | 18.1-28.9 | 0 |
| *Swelling* | 33.7 | 29.8-37.8 | 16.15 | 11.4-22.4 | 0 | 31.5 | 26.6-36.8 | 18.4 | 13.9-23.9 | 0.0006 |
| Lip/tongue swelling | 42.1 | 37.9-46.3 | 13.4 | 9.6-18.6 | 0 | 44.7 | 39.1-50.48 | 21.2 | 16.9-26.2 | 0 |
| *Difficulty swallowing* | 47 | 42.78-51.3 | 17.7 | 13.4-23.1 | 0 | 51.1 | 45.3-56.9 | 20.5 | 15.8-26.1 | 0 |
| Hoarse voice | 18.4 | 15.2-22.2 | 11.1 | 7.1-16.9 | 0.0298 | 26.3 | 21.5-31.7 | 13.1 | 9.4-17.8 | 0.0001 |
| Itchy mouth | 32.7 | 28.9-36.7 | 14.5 | 10.1-20.3 | 0 | 52.5 | 46.8-58.2 | 23.2 | 18.5-28.7 | 0 |
| *Throat tightening* | 37.05 | 33.0-41.3 | 20 | 14.9-26.2 | 0 | 46.1 | 40.4-51.9 | 22.1 | 17.3-28.0 | 0 |
| Mouth or throat tingling | 20.11 | 16.8-23.9 | 14.5 | 10.4-20 | 0.0824 | 30.78 | 25.6-36.5 | 16.9 | 13.0-21.5 | 0.0001 |
| **Respiratory** |  |  |  |  |  |  |  |  |  |  |
| **Any Stringent Respiratory** | 60.1 | 55.9-64.1 | 56.4 | 47.2-65.2 | 0.47 | 55.7 | 49.9-61.4 | 57.9 | 49.3-66.0 | 0.68 |
| *Chest tightening* | 31 | 27.2-35.1 | 13.5 | 9.4-19.1 | 0 | 30.44 | 25.4-36.0 | 14.3 | 10.2-19.5 | 0 |
| Nasal congestion | 18.8 | 16.0-22.0 | 12.9 | 8.9-18.6 | 0.0636 | 21.4 | 17.1-26.4 | 9.2 | 6.7-12.4 | 0 |
| Repetitive cough | 16 | 12.7-19.9 | 12 | 8.1-17.5 | 0.2109 | 21.4 | 16.4-27.4 | 7.9 | 5.2-11.8 | 0 |
| *Trouble breathing* | 33.7 | 29.8-17.9 | 16.4 | 12.1-22 | 0 | 35.9 | 30.7-41.5 | 16 | 12.3-20.4 | 0 |
| *Wheezing* | 25.1 | 21.5-29.1 | 9.6 | 6.7-13.6 | 0 | 26.73 | 22-32.1 | 14.8 | 10.6-20.4 | 0.0017 |
| **Gastrointestinal** |  |  |  |  |  |  |  |  |  |  |
| **Any Stringent GI** | 12.2 | 9.3-15.8 | 3.3 | 1.6-6.5 | 0.0002 | 15.8 | 12.4-19.9 | 5.9 | 3.9-8.9 | 0 |
| Belly pain | 18.9 | 15.9-22.3 | 13.2 | 9.5-18.1 | 0.048 | 25.12 | 20.3-30.7 | 11.3 | 7.7-16.3 | 0.0001 |
| Cramps | 19.3 | 15.9-23.2 | 11.9 | 8.5-16.4 | 0.0103 | 26.4 | 21.4-32.1 | 9.2 | 6.6-12.6 | 0 |
| Diarrhea | 16.5 | 13.2-20.3 | 9.2 | 6.1-13.8 | 0.0115 | 20.82 | 16.3-26.3 | 10.2 | 6.7-15.3 | 0.0025 |
| Nausea | 17 | 14.2-20.3 | 8.8 | 5.8-13.1 | 0.0024 | 25.1 | 20.1-30.9 | 8.1 | 5.7-11.2 | 0 |
| *Vomiting* | 12.2 | 9.3-15.8 | 3.3 | 1.6-6.5 | 0.0002 | 15.8 | 12.4-19.9 | 5.9 | 3.9-8.9 | 0 |
| **Cardiovascular** |  |  |  |  |  |  |  |  |  |  |
| **Any Stringent CV** | 45.1 | 40.8-49.4 | 46.5 | 37.5-55.7 | 0.79 |  |  |  |  |  |
| *Chest pain* | 15.1 | 12.3-18.5 | 4.3 | 2.7-6.8 | 0 | 16.1 | 12.6-20.3 | 5.8 | 4.0-8.4 | 0 |
| *Rapid heart rate* | 18.8 | 15.8-22.0 | 11.2 | 7.7-16 | 0.0083 | 18 | 14.4-22.3 | 9 | 6.5-12.3 | 0.0003 |
| *Fainting, dizziness, or feeling light headed* | 19.9 | 16.3-24.0 | 8.3 | 5.6-12.2 | 0 | 22.3 | 17.3-28.2 | 8.5 | 6.0-11.9 | 0 |
| *Low blood pressure* | 6.4 | 4.5-9.0 | 3.8 | 2.1-6.7 | 0.1218 | 6.6 | 3.8-11.3 | 3.2 | 1.8-5.5 | 0.057 |

|  | | **Symptom Characteristics of Adults with Childhood-Onset vs. Adult-Onset Milk Allergy, Population-weighted Frequency % (95% CI)** | | | | | **Symptom Characteristics of Adults with Childhood-Onset vs. Adult-Onset Egg Allergy, Population-weighted Frequency % (95% CI)** | | | | |
| --- | --- | --- | --- | --- | --- | --- | --- | --- | --- | --- | --- |
|  |  | | |  | |  |  | |  | |  |
| **Symptoms** | **Childhood Onset Milk Allergy** | | | **Adult Onset Milk Allergy** | | **p value** | **Childhood Onset Egg Allergy** | | **Adult Onset Egg Allergy** | | **p value** |
|  | **Point Estimate** | | **(95% CI)** | **Point Estimate** | **(95% CI)** |  | **Point Estimate** | **(95% CI)** | **Point Estimate** | **(95% CI)** |  |
| **Skin/Oral/Mucosal Tissue** |  | |  |  |  |  |  |  |  |  |  |
| **Any *Stringent* Skin** |  | |  |  |  |  |  |  |  |  |  |
| *Hives* | 35.4 | | 30.6-40.5 | 5.3 | 3.9-7.2 | 0 | 45.8 | 37.6-54.3 | 14.5 | 9.3-21.9 | 0 |
| Itching | 36.5 | | 31.2-41.3 | 5.4 | 3.9-7.3 | 0 | 52.3 | 44.1-60.5 | 17.9 | 12.3-25.3 | 0 |
| *Rash* | 1.5 | | 0.8-2.6 | 0.2 | 0.03-0.7 | 0.0005 | 33.4 | 27-40.7 | 9.6 | 6.3-14.6 | 0 |
| *Swelling* | 0.6 | | 0.3-1.4 | 0.4 | 0.05-2.6 | 0.5899 | 18.8 | 14.2-24.5 | 6.4 | 3.8-10.7 | 0.0001 |
| Lip/tongue swelling | 16.6 | | 13.4-20.3 | 2.6 | 1.7-4.1 | 0 | 20.7 | 14.3-29.1 | 5.4 | 3.0-9.5 | 0 |
| *Difficulty* *swallowing* | 20.2 | | 16.7-24.2 | 3.9 | 2.6-5.8 | 0 | 26.7 | 18.8-36.4 | 9.4 | 6.1-14.1 | 0.0001 |
| Hoarse voice | 9.8 | | 7.6-12.6 | 2.2 | 1.3-3.7 | 0 | 10.1 | 6.9-14.5 | 3.7 | 1.7-8 | 0.0175 |
| Itchy mouth | 15.8 | | 12.9-19.2 | 3.3 | 2.3-4.9 | 0 | 22 | 17.2-27.8 | 4.8 | 2.9-7.8 | 0 |
| *Throat tightening* | 16.9 | | 13.7-20.6 | 3.5 | 2.2-5.4 | 0 | 31.3 | 16.2-27.5 | 3.9 | 2.2-7.0 | 0 |
| Mouth or throat tingling | 13 | | 9,4-17.8 | 2.3 | 1.4-3.7 | 0 | 12.7 | 9.0-17.5 | 4.1 | 2.3-7.0 | 0.0003 |
| **Respiratory** |  | |  |  |  |  |  |  |  |  |  |
| **Any Stringent Respiratory** |  | |  |  |  |  |  |  |  |  |  |
| *Chest tightening* | 14 | | 11.0-17.6 | 2.5 | 1.4-4.5 | 0 | 10.3 | 7.0-14.8 | 4.1 | 2.0-8.2 | 0.019 |
| Nasal congestion | 21.4 | | 17.1-26.3 | 4.5 | 3.2-6.3 | 0 | 13.7 | 9.3-19.8 | 2.6 | 1.4-4.8 | 0 |
| Repetitive cough | 12.6 | | 9.8-16.2 | 3.1 | 1.9-4.9 | 0 | 8.8 | 5.8-13.0 | 4.5 | 2.5-7.9 | 0.0551 |
| *Trouble breathing* | 14.7 | | 11.4-18.7 | 3.2 | 2.1-5.0 | 0 | 16.5 | 9.5-27.1 | 6.1 | 3.9-9.5 | 0.0043 |
| *Wheezing* | 15.5 | | 12.3-19.2 | 4.2 | 2.8-6.5 | 0 | 11 | 7.6-15.8 | 4.1 | 2.2-7.4 | 0.0043 |
| **Gastrointestinal** |  | |  |  |  |  |  |  |  |  |  |
| **Any Stringent GI** | 39.7 | | 34.9-44.8 | 8.9 | 6.9-11.3 | 0 | 31.3 | 23.25-40.7 | 17.1 | 11.6-24.5 | 0.01 |
| Belly pain | 56.7 | | 51.9-61.3 | 16.3 | 13.4-19.7 | 0 | 27.3 | 19.6-36.7 | 14.2 | 10.1-19.7 | 0.0052 |
| Cramps | 52.3 | | 47.4-57.1 | 16.5 | 13.5-20.1 | 0 | 25.2 | 19.7-31.6 | 13.3 | 9.1-18.9 | 0.003 |
| Diarrhea | 60.3 | | 55.5-64.9 | 15.3 | 12.4-19.7 | 0 | 28.9 | 21.0-38.4 | 12.2 | 8.4-17.5 | 0.0003 |
| Nausea | 43.6 | | 38.7-48.6 | 10.5 | 8.2-13.2 | 0 | 33.6 | 25.2-43.1 | 15.9 | 10.7-23.11 | 0.0014 |
| *Vomiting* | 39.7 | | 34.9-44.8 | 8.9 | 6.9-11.3 | 0 | 31.3 | 23.25-40.7 | 17.1 | 11.6-24.5 | 0.01 |
| **Cardiovascular** |  | |  |  |  |  |  |  |  |  |  |
| **Any Stringent CV** |  | |  |  |  |  |  |  |  |  |  |
| *Chest pain* | 6.8 | | 4.9-9.4 | 1.2 | 0.5-2.4 | 0 | 5.6 | 3.4-9.0 | 1.4 | 0.6-3.5 | 0.0052 |
| *Rapid heart rate* | 9.8 | | 7.6-12.6 | 2 | 1.1-3.7 | 0 | 10.5 | 7.3-15.0 | 3.2 | 1.8-5.7 | 0.0003 |
| *Fainting, dizziness, or feeling light headed* | 10.36 | | 7.9-13.5 | 3.7 | 2.4-5.8 | 0.0001 | 11.5 | 7.6-17.1 | 3 | 1.5-5.9 | 0.0004 |
| *Low blood pressure* | 4.6 | | 2.9-7.1 | 1.1 | 0.5-2.3 | 0.0003 | 4.2 | 2.3-7.7 | 2.3 | 1.0-5.2 | 0.242 |

|  | **Symptom Characteristics of Adults with Childhood-Onset vs. Adult-Onset Shellfish Allergy, Population-weighted Frequency % (95% CI)** | | | | | **Symptom Characteristics of Adults with Childhood-Onset vs. Adult-Onset Fin fish Allergy, Population-weighted Frequency % (95% CI)** | | | | |
| --- | --- | --- | --- | --- | --- | --- | --- | --- | --- | --- |
| **Symptoms** | **Childhood Onset Shellfish Allergy** | | **Adult Onset Shellfish Allergy** | | **p value** | **Childhood Onset Fin fish Allergy** | | **Adult Onset Fin fish Allergy** | | **p value** |
|  | **Point Estimate** | **(95% CI)** | **Point Estimate** | **(95% CI)** |  | **Point Estimate** | **(95% CI)** | **Point Estimate** | **(95% CI)** |  |
| **Skin/Oral/Mucosal Tissue** |  |  |  |  |  |  |  |  |  |  |
| **Any *Stringent* Skin** |  |  |  |  |  |  |  |  |  |  |
| *Hives* | 68.7 | 64.4-72.7 | 43.2 | 39.0-47.5 | 0 | 63.8 | 56.4-70.5 | 38.7 | 31.5-26.5 | 0 |
| Itching | 70.1 | 65.8-74.1 | 42.7 | 38.5-47.0 | 0 | 70.7 | 64.1-76.5 | 47.8 | 39.8-56.0 | 0 |
| *Rash* | 53.5 | 49.0-57.9 | 31.3 | 27.4-35.5 | 0 | 52.6 | 45.5-59.6 | 39.1 | 317-47.0 | 0.0125 |
| *Swelling* | 37 | 32.7-41.6 | 23.4 | 20.1-27.1 | 0 | 32 | 25.3-39.5 | 22.6 | 16.7-29.7 | 0.0584 |
| Lip/tongue swelling | 40.5 | 36.1-45 | 24.7 | 21.2-28.6 | 0 | 39.1 | 32.2-46.6 | 28.4 | 21.8-36.0 | 0.0411 |
| *Difficulty swallowing* | 42.5 | 38.1-47.1 | 25.2 | 21.8-28.9 | 0 | 35.7 | 29.4-42.6 | 29.7 | 23.1-37.4 | 0.2353 |
| Hoarse voice | 18.8 | 15.5-22.6 | 10.4 | 8.1-13.3 | 0.0001 | 25.8 | 19.8-33.0 | 16.5 | 11.5-23.2 | 0.0401 |
| Itchy mouth | 38.7 | 34.3-43.3 | 18.8 | 15.9-22.1 | 0 | 38.3 | 31.4-45.7 | 26 | 19.9-33.4 | 0.0168 |
| *Throat tightening* | 37.8 | 33.5-42.3 | 23.5 | 20.3-27.0 | 0 | 35.4 | 28.8-42.6 | 26.1 | 19.9-33.3 | 0.0618 |
| Mouth or throat tingling | 27 | 22.9-31.5 | 14.6 | 11.9-17.9 | 0 | 18.5 | 13.5-24.8 | 15.7 | 11.3-21.6 | 0.4762 |
| **Respiratory** |  |  |  |  |  |  |  |  |  |  |
| **Any Stringent Respiratory** |  |  |  |  |  |  |  |  |  |  |
| *Chest tightening* | 26.5 | 22.5-30.9 | 13.3 | 10.9-16.1 | 0 | 25.6 | 19.3-33.1 | 16.4 | 11.4-23 | 0.0462 |
| Nasal congestion | 16.8 | 12.9-20.1 | 9.4 | 7.4-11.9 | 0.0001 | 19 | 14.0-25.3 | 9.9 | 6.5-14.8 | 0.0107 |
| Repetitive cough | 14.9 | 11.9-18.5 | 7.5 | 5.7-9.8 | 0.0001 | 17.7 | 12.8-24.0 | 10.6 | 6.7-16.0 | 0.0536 |
| *Trouble breathing* | 33.3 | 29.2-37.6 | 21.5 | 18.2-25.2 | 0 | 31.5 | 24.2-38.6 | 26.2 | 20.0-33.6 | 0.2804 |
| *Wheezing* | 21 | 17.5-24.9 | 11.8 | 9.3-14.9 | 0.0001 | 18.6 | 13.9-24.3 | 12.9 | 8.8-18.7 | 0.1222 |
| **Gastrointestinal** |  |  |  |  |  |  |  |  |  |  |
| **Any Stringent GI** | 28.3 | 24.4-32.5 | 22.5 | 19.1-26.3 | 0.0359 | 28.6 | 22.2-35.7 | 24.5 | 18.6-31.6 | 0.3936 |
| Belly pain | 25.1 | 31.4-29.2 | 17.2 | 14.3-20.5 | 0.0018 | 21.7 | 16.2-28.6 | 20.8 | 15.3-27.7 | 0.8359 |
| Cramps | 24.2 | 20.5-28.4 | 17.7 | 14.8-20.9 | 0.009 | 25 | 18.7-32.5 | 19.5 | 14.3-26.0 | 0.2311 |
| Diarrhea | 24.6 | 20.7-28.9 | 14.8 | 12.2-17.7 | 0.0001 | 18.6 | 14.0-24.4 | 21.1 | 15.5-28.1 | 0.5401 |
| Nausea | 32.9 | 28.6-37.4 | 24.8 | 21.2-28.7 | 0.0057 | 30.8 | 24.4-37.9 | 31.7 | 24.9-39.3 | 0.857 |
| *Vomiting* | 28.3 | 24.4-32.5 | 22.5 | 19.1-26.3 | 0.0359 | 28.6 | 22.2-35.7 | 24.5 | 18.6-31.6 | 0.3936 |
| **Cardiovascular** |  |  |  |  |  |  |  |  |  |  |
| **Any Stringent CV** |  |  |  |  |  |  |  |  |  |  |
| *Chest pain* | 13.5 | 10.7-16.9 | 4 | 2.6-6.0 | 0 | 16.1 | 10.6-23.7 | 6.4 | 3.8-10.5 | 0.0039 |
| *Rapid heart rate* | 19.3 | 16-23.2 | 11.4 | 9.1-14.4 | 0.0004 | 22.2 | 16.6-29.0 | 16.5 | 11.5-23.2 | 0.194 |
| *Fainting, dizziness, or feeling light headed* | 17.4 | 13.9-21.5 | 13.3 | 10.7-16.3 | 0.0841 | 13.8 | 10.2-18.4 | 18 | 13-24.5 | 0.2283 |
| *Low blood pressure* | 5.9 | 4.2-8.2 | 2 | 1.3-3.1 | 0.0001 | 9.5 | 5.5-15.8 | 4.1 | 1.9-8.4 | 0.0615 |

|  | **Symptom Characteristics of Adults with Childhood-Onset vs. Adult-Onset Wheat Allergy, Population-weighted Frequency % (95% CI)** | | | | | **Symptom Characteristics of Adults with Childhood-Onset vs. Adult-Onset Soy Allergy, Population-weighted Frequency % (95% CI)** | | | | |
| --- | --- | --- | --- | --- | --- | --- | --- | --- | --- | --- |
| **Symptoms** | **Childhood Onset Wheat Allergy** | | **Adult Onset Wheat Allergy** | | **p value** | **Childhood Onset Soy Allergy** | | **Adult Onset Soy Allergy** | | **p value** |
|  | **Point Estimate** | **(95% CI)** | **Point Estimate** | **(95% CI)** |  | **Point Estimate** | **(95% CI)** | **Point Estimate** | **(95% CI)** |  |
| **Skin/Oral/Mucosal Tissue** |  |  |  |  |  |  |  |  |  |  |
| **Any *Stringent* Skin** |  |  |  |  |  |  |  |  |  |  |
| *Hives* | 49.1 | 40.7-57.5 | 8.6 | 6.4-11.5 | 0 | 54.5 | 45.3-63.4 | 20.7 | 15.9-26.6 | 0 |
| Itching | 47.6 | 39.3-56.1 | 12.6 | 9.2-17 | 0 | 45 | 36.1-54.3 | 22 | 16.7-28.1 | 0 |
| *Rash* | 2.4 | 1.0-5.3 | 0.7 | 0.3-1.9 | 0.053 | 38.1 | 29.7-47.3 | 12.9 | 9.2-17.8 | 0 |
| *Swelling* | 1 | 0.2-4.0 | 0 |  | 0.0117 | 12.2 | 8.1-17.9 | 7.4 | 4.8-11.4 | 0.0953 |
| Lip/tongue swelling | 12 | 8.1-17.4 | 3.3 | 1.9-5.8 | 0.0001 | 20.8 | 14.2-29.2 | 3.8 | 1.9-7.2 | 0 |
| *Difficulty swallowing* | 17.3 | 12.4-23.7 | 5 | 2.6-9.3 | 0.0002 | 25.5 | 17.7-32.9 | 6.7 | 3.9-11.2 | 0 |
| Hoarse voice | 15.2 | 9.1-24.4 | 4.5 | 2.7-7.5 | 0.0005 | 15.1 | 10.2-21.9 | 4.3 | 2.4-7.6 | 0.0001 |
| Itchy mouth | 20.8 | 14.5-29.7 | 5 | 2.6-9.4 | 0 | 27.1 | 18.6-37.8 | 9.9 | 6.6-14.5 | 0.0002 |
| *Throat tightening* | 15.6 | 11.8-25.4 | 4.3 | 2.6-7.1 | 0 | 23.3 | 15.9-32.9 | 6.6 | 4.2-10.25 | 0 |
| Mouth or throat tingling | 6.7 | 4.2-10.6 | 4.5 | 2.3-8.6 | 0.3261 | 15.8 | 9.6-24.8 | 6 | 3.6-9.8 | 0.0049 |
| **Respiratory** |  |  |  |  |  |  |  |  |  |  |
| **Any Stringent Respiratory** |  |  |  |  |  |  |  |  |  |  |
| *Chest tightening* | 13.7 | 9.4-19.4 | 4.6 | 2.8-7.2 | 0.0001 | 13.8 | 9.4-19.9 | 5.6 | 2.9-10.4 | 0.0126 |
| Nasal congestion | 16.6 | 10.9-22 | 7.3 | 4.5-11.7 | 0.0098 | 25.5 | 17.5-35.5 | 5.4 | 3.3-8.6 | 0 |
| Repetitive cough | 17.1 | 10.1-27.4 | 2.9 | 2.5-9.2 | 0.0016 | 18.9 | 11.o-30.4 | 4.9 | 2.9-8.1 | 0.0001 |
| *Trouble breathing* | 25 | 18-33.6 | 7.5 | 4.8-11.7 | 0 | 18.8 | 13.4-25.8 | 7.1 | 4.5-11.2 | 0.0004 |
| *Wheezing* | 13.2 | 8.4-20.1 | 5.7 | 3.6-9 | 0.0086 | 13.4 | 8.6-20.3 | 8.7 | 5.7-13.1 | 0.1561 |
| **Gastrointestinal** |  |  |  |  |  |  |  |  |  |  |
| **Any Stringent GI** | 18.8 | 14.4-26.5 | 13.6 | 9.7-18.8 | 0.1025 | 19.5 | 13.9-26.7 | 12.6 | 8.5-18.3 | 0.085 |
| Belly pain | 41.3 | 33.3-50 | 21 | 16.5-26.3 | 0 | 26.8 | 19.5-35.6 | 13.8 | 9.7-19.5 | 0.0048 |
| Cramps | 40.7 | 32.6-49.3 | 18.2 | 14-23.4 | 0 | 24 | 16.9-32.9 | 15.2 | 10.9-20.8 | 0.0551 |
| Diarrhea | 33.6 | 26.5-41.6 | 17.4 | 13.3-22.6 | 0.0002 | 27.3 | 20.2-35.9 | 16 | 11.7-21.5 | 0.0122 |
| Nausea | 36.9 | 28.6-46 | 15.3 | 11.3-20.5 | 0 | 29.5 | 20.9-40 | 13.6 | 9.5-19.2 | 0.0015 |
| *Vomiting* | 18.8 | 14.4-26.5 | 13.6 | 9.7-18.8 | 0.1025 | 19.5 | 13.9-26.7 | 12.6 | 8.5-18.3 | 0.085 |
| **Cardiovascular** |  |  |  |  |  |  |  |  |  |  |
| **Any Stringent CV** |  |  |  |  |  |  |  |  |  |  |
| *Chest pain* | 7.2 | 4.4-11.6 | 2.8 | 1.0-7.4 | 0.0794 | 8.3 | 5.1-13.3 | 1.7 | 0.8-3.8 | 0.0003 |
| *Rapid heart rate* | 15.8 | 11.2-21.9 | 6.7 | 4.2-10.6 | 0.0026 | 13 | 9.0-18.5 | 8 | 7.4-12.8 | 0.0795 |
| *Fainting, dizziness, or feeling light headed* | 17.9 | 11.1-27.6 | 7.5 | 4.6-11.8 | 0.0075 | 16.7 | 9.7-27.3 | 8.8 | 5.6-13.6 | 0.0647 |
| *Low blood pressure* | 6.1 | 3.4-10.7 | 1.6 | 0.7-3.6 | 0.0051 | 7.4 | 3.8-13.8 | 2.2 | 1.0-5.1 | 0.0178 |

|  | **Symptom Characteristics of Adults with Childhood-Onset vs. Adult-Onset Sesame Allergy, Population-weighted Frequency % (95% CI)** | | | | |
| --- | --- | --- | --- | --- | --- |
| **Symptoms** | **Childhood Onset Sesame Allergy** | | **Adult Onset Sesame Allergy** | | **p value** |
|  | **Point Estimate** | **(95% CI)** | **Point Estimate** | **(95% CI)** |  |
| **Skin/Oral/Mucosal Tissue** |  |  |  |  |  |
| **Any *Stringent* Skin** |  |  |  |  |  |
| *Hives* | 69.5 | 58.4-78.8 | 35 | 23.3-28.7 | 0.0002 |
| Itching | 51.5 | 38.8-64.1 | 26.5 | 16.5-39.6 | 0.0068 |
| *Rash* | 23 | 14.9-33.6 | 19.9 | 11.4-32.4 | 0.6735 |
| *Swelling* | 13 | 6.7-23.8 | 10.6 | 4.9-21.4 | 0.6793 |
| Lip/tongue swelling | 29.32 | 17.3-45.2 | 10.9 | 5.8-19.3 | 0.009 |
| *Difficulty swallowing* | 18.2 | 11.4-27.7 | 15.6 | 7.8-28.7 | 0.6937 |
| Hoarse voice | 13.1 | 6.8-23.6 | 10.9 | 5.3-21.2 | 0.7091 |
| Itchy mouth | 17.7 | 10.5-28.1 | 12.1 | 5.9-23.1 | 0.3735 |
| *Throat tightening* | 20.35 | 12.7-31.0 | 8.9 | 3.9-19.5 | 0.0665 |
| Mouth or throat tingling | 8.3 | 4.1-16.3 | 6 | 2.7-12.8 | 0.5448 |
| **Respiratory** |  |  |  |  |  |
| **Any Stringent Respiratory** |  |  |  |  |  |
| *Chest tightening* | 16.4 | 9.7-26.4 | 6 | 2.2-15.4 | 0.0604 |
| Nasal congestion | 10.8 | 5.5-20.2 | 10.6 | 4.4-23.5 | 0.969 |
| Repetitive cough | 6.1 | 3.0-12 | 5.3 | 1.4-17.6 | 0.8314 |
| *Trouble breathing* | 28.5 | 19-40.4 | 7.6 | 2.5-20.5 | 0.0085 |
| *Wheezing* | 7.5 | 3.5-15.0 | 3.4 | 1.0-10.6 | 0.2455 |
| **Gastrointestinal** |  |  |  |  |  |
| **Any Stringent GI** | 5.6 | 2.4-12.4 | 8.2 | 3.0-20.9 | 0.5447 |
| Belly pain | 9.3 | 4.9-17 | 13.5 | 6.3-16.6 | 0.438 |
| Cramps | 12.4 | 6.7-21.8 | 16.3 | 8.0-30.3 | 0.5509 |
| Diarrhea | 10.3 | 5.2-19.3 | 8.4 | 3.6-18.1 | 0.6965 |
| Nausea | 17.6 | 10.1-28.9 | 12.6 | 5.5-26.1 | 0.4914 |
| *Vomiting* | 5.6 | 2.4-12.4 | 8.2 | 3.0-20.9 | 0.5447 |
| **Cardiovascular** |  |  |  |  |  |
| **Any Stringent CV** |  |  |  |  |  |
| *Chest pain* | 14.8 | 8.2-25.2 | 2.7 | 0.4-17.1 | 564 |
| *Rapid heart rate* | 10.1 | 4.8-19.9 | 15.1 | 7.1-19.2 | 0.4311 |
| *Fainting, dizziness, or feeling light headed* | 9 | 4.5-17.2 | 13.7 | 6.3-17.1 | 0.4013 |
| *Low blood pressure* | 8.3 | 3.5-18.4 | 1.3 | 0.4-4.15 | 0.0041 |

**eTable 2. Prevalence of Comorbid atopic conditions among adults with specific childhood- or adult-onset food allergies, Population-weighted Frequency % (95% CI)**

| **Variable** | **Adults with Childhood-onset Peanut Allergy** | | **Adults with Adult-onset Peanut Allergy** | |  | **Adults with Childhood-onset Tree Nut Allergy** | | **Adults with Adult-onset Tree Nut Allergy** | |  | **Adults with Childhood-onset Milk Allergy** | | **Adults with Adult-onset Milk Allergy** | |  |
| --- | --- | --- | --- | --- | --- | --- | --- | --- | --- | --- | --- | --- | --- | --- | --- |
| ***Physician Diagnosed Comorbid Conditions*** | Point Estimate | 95% CI | Point Estimate | 95% CI | p-value | Point Estimate | 95% CI | Point Estimate | 95% CI | p-value | Point Estimate | 95% CI | Point Estimate | 95% CI | p-value |
| **Asthma** | 24.23 | 20.9-28.0 | 28.8 | 21.1-37.9 | 0.31 | 23.51 | 19.3-28.3 | 28.7 | 21.8-36.7 | 0.23 | 28.49 | 24.0-33.5 | 21.5 | 15.3-29.2 | 0.12 |
| **Atopic Dermatitis/Eczema** | 12.9 | 10.4-16 | 9.5 | 5.7-15.5 | 0.27 | 16.1 | 12.0-21.1 | 16.5 | 11.4-23.4 | 0.91 | 18.41 | 15.1-22.3 | 8.3 | 5.2-12.9 | 0.0008 |
| **Environmental Allergies** | 23.4 | 19.8-27.4 | 37.8 | 29-3-47.2 | 0.002 | 27.8 | 23.1-33.1 | 47 | 38.5-55.6 | *0.0001* | 33.2 | 29.0-37.7 | 42.5 | 34.4-51.1 | 0.049 |
| **Insect Sting Allergy** | 5.1 | 3.2-7.8 | 9.3 | 5.1-16.4 | 0.11 | 7.5 | 4.8-11.5 | 8.4 | 4.9-14.2 | 0.74 | 7.9 | 5.8-10.7 | 9.6 | 5.4-16.7 | 0.55 |
| **Latex Allergy** | 3.6 | 2.4-5.3 | 10.7 | 5.3-20.6 | 0.005 | 5.2 | 3.2-8.3 | 13.7 | 8.0-22.3 | 0.006 | 7.5 | 5.4-10.4 | 12.3 | 7.2-20.3 | 0.12 |
| **Medication Allergy** | 10.4 | 7.9-13.6 | 19.4 | 13.4-27.2 | 0.007 | 13.7 | 10.3-18.1 | 23.3 | 17.1-30.9 | 0.01 | 19.2 | 16-23 | 26.8 | 20.1-34.6 | 0.05 |

| **Variable** | **Adults with Childhood-onset Egg Allergy** | | **Adults with Adult-onset Egg Allergy** | |  | **Adults with Childhood-onset Shellfish Allergy** | | **Adults with Adult-onset Shellfish Allergy** | |  | **Adults with Childhood-onset Fin Fish Allergy** | | **Adults with Adult-onset Fin Fish Allergy** | |  |
| --- | --- | --- | --- | --- | --- | --- | --- | --- | --- | --- | --- | --- | --- | --- | --- |
| ***Physician Diagnosed Comorbid Conditions*** | Point Estimate | 95% CI | Point Estimate | 95% CI | p-value | Point Estimate | 95% CI | Point Estimate | 95% CI | p-value | Point Estimate | 95% CI | Point Estimate | 95% CI | p-value |
| **Asthma** | 31.16 | 23.3-40.3 | 23 | 14.7-34.0 | 0.23 | 23.06 | 19.7-26.8 | 23.3 | 19.3-27.7 | 0.94 | 25.3 | 19.7-31.9 | 25.1 | 18.1-33.7 | 0.97 |
| **Atopic Dermatitis/Eczema** | 13.1 | 7.6-21.8 | 9.6 | 5.3-16.6 | 0.43 | 11.2 | 8.7-14.4 | 12.2 | 9.2-16.1 | 0.66 | 9.3 | 5.9-14.4 | 10.1 | 6.0-16.5 | 0.81 |
| **Environmental Allergies** | 23.3 | 17.9-29.7 | 32.5 | 22.8-44.0 | 0.12 | 25.1 | 21.7-28.9 | 42.4 | 37.3-47.6 | <.0001 | 21.7 | 16.7-27.6 | 34.2 | 26.2-43.3 | 0.01 |
| **Insect Sting Allergy** | 5.4 | 3.1-9.3 | 3.6 | 1.4-9.1 | 0.46 | 6.4 | 4.8-8.5 | 8.9 | 6.6-12.0 | 0.11 | 6.3 | 3.4-11.2 | 15.7 | 10.4-23.1 | 0.009 |
| **Latex Allergy** | 8.1 | 5.1-12.4 | 6.2 | 3.1-12.0 | 0.52 | 4.6 | 3.4-6.3 | 6.2 | 4.2-9.0 | 0.25 | 3.8 | 2.0-7.3 | 9.2 | 5.5-15.0 | 0.03 |
| **Medication Allergy** | 17.7 | 13-23.6 | 16.4 | 9.9-25.9 | 0.79 | 17.8 | 14.6-21.6 | 30.9 | 26.3-35.8 | 0.0004 | 13.6 | 9.5-19.2 | 29.3 | 21.9-38.0 | 0.0007 |

| **Variable** | **Adults with Childhood-onset Wheat Allergy** | | **Adults with Adult-onset Wheat Allergy** | |  | **Adults with Childhood-onset Soy Allergy** | | **Adults with Adult-onset Soy Allergy** | |  | **Adults with Childhood-onset Sesame Allergy** | | **Adults with Adult-onset Sesame Allergy** | |  |
| --- | --- | --- | --- | --- | --- | --- | --- | --- | --- | --- | --- | --- | --- | --- | --- |
| ***Physician Diagnosed Comorbid Conditions*** | Point Estimate | 95% CI | Point Estimate | 95% CI | p-value | Point Estimate | 95% CI | Point Estimate | 95% CI | p-value | Point Estimate | 95% CI | Point Estimate | 95% CI | p-value |
| **Asthma** | 30.32 | 23.6-38.1 | 21.5 | 15.0-29.9 | 0.1 | 22.4 | 16.5-29.7 | 35.9 | 26.8-46.0 | 0.02 | 26.9 | 17.5-38.9 | 20.4 | 10.0-37.2 | 0.48 |
| **Atopic Dermatitis/Eczema** | 13.9 | 9.4-20.2 | 10.4 | 6.5-16.1 | 0.33 | 9.6 | 5.9-15.3 | 17.2 | 10.7-26.4 | 0.08 | 11.9 | 6.4-20.9 | 10.9 | 3.5-29.2 | 0.9 |
| **Environmental Allergies** | 31.6 | 24.3-40.0 | 47 | 37.3-56.9 | *0.02* | 17.7 | 12.6-24.2 | 51.6 | 41.6-61.5 | <.0001 | 17.5 | 10.3-28.3 | 40 | 23.6-58.9 | 0.02 |
| **Insect Sting Allergy** | 10.2 | 5.9-17.1 | 8.7 | 5.1-14.4 | 0.67 | 5.2 | 2.6-9.8 | 18.3 | 11.2-28.5 | 0.001 | 6.2 | 2.4-14.8 | 4.7 | 1.3-15.9 | 0.74 |
| **Latex Allergy** | 10 | 6.2-15.7 | 7.3 | 4.1-12.5 | 0.38 | 7.4 | 3.6-14.5 | 15.4 | 9.1-24.9 | 0.09 | 9.1 | 4.4-10.3 | 4.7 | 1.0-19.2 | 0.41 |
| **Medication Allergy** | 17.9 | 12.2-25.4 | 27.7 | 20.3-36.7 | 0.07 | 11.8 | 7.6-17.9 | 31.9 | 23.3-42.0 | 0.0001 | 15.4 | 5.9-34.5 | 27.2 | 13.9-46.5 | 0.29 |
